# Nanoscale Filament Evolution and Local Resistive Switching in Substoichiometric Yttrium Oxide Thin Films

**DOI:** 10.1021/acs.nanolett.6c04262

**Published:** 2026-09-28

**Authors:** Yen-Po Liu, Erkai Wang, Philipp Schreyer, Yu Duan, Luisa Bayer, Yingxin Li, Taewook Kim, Alexey Arzumanov, Stefan Wiefels, Eszter Piros, Lambert Alff, Regina Dittmann

**Affiliations:** † Peter Grünberg Institute (PGI-7), 28334 Forschungszentrum Jülich GmbH, 52425 Jülich, Germany; ‡ Advanced Thin Film Technology Division, Institute of Materials Science, 26536 TU Darmstadt, 64287 Darmstadt, Germany

**Keywords:** Yttrium oxide, Resistive switching, Memristors, Analog switching, Filament evolution, Conductive atomic force microscopy

## Abstract

Scaling resistive random-access memory (RRAM) toward ultradense crossbar arrays for high-density storage and neuromorphic computing requires precise nanoscale control of conductive filaments for reliable multilevel operation. Here we directly visualize and modulate nanoscale filament evolution in substoichiometric Y_2_O_3–*x*_ thin films using high-resolution *in situ* conductive atomic force microscopy (c-AFM) with controlled current compliance. The switching threshold systematically increases with increasing oxygen stoichiometry, while filament evolution is continuously modulated by varying the applied bias. The initially formed larger filaments persist and dominate subsequent switching events during multicycle measurements. Using the c-AFM tip as a nanoscale mobile top electrode, single-point switching reveals localized switching with a lateral influence of approximately 20 nm, defining a crosstalk length scale. These results directly link oxygen stoichiometry, microstructure, filament evolution, and switching length scales, providing new insight into nanoscale filamentary switching and physical guidelines for scaling polycrystalline memristors toward ultradense memory and neuromorphic applications.

## Graphical abstract

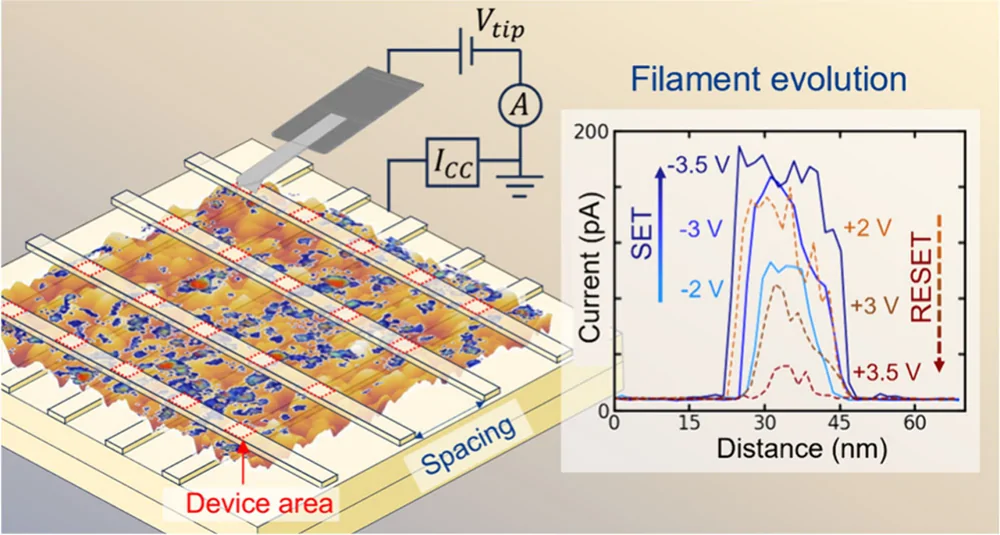

Yttrium oxide (Y_2_O_3_) is a promising candidate for resistive random-access memory (RRAM) because of its exceptional thermodynamic stability,^1–3^ high dielectric constant (*k* ∼ 15),^4–7^ and compatibility with complementary metal–oxide–semiconductor (CMOS) processes.^8,9^ Valence change memory (VCM) operates by modulating the resistance of an insulating oxide layer through voltage-driven oxygen vacancy redistribution, leading to the formation and dissolution of conductive filaments, thereby switching between high and low resistive states (HRS and LRS). Y_2_O_3_ in its body-centered cubic phase has a natural tendency toward 25% oxygen defects and is widely reported to exhibit forming-free behavior.^9–13^ Eliminating electroforming reduces voltage overhead and simplifies postfabrication testing, offering a key advantage for scalable and energy-efficient memristor technologies. In addition, its polycrystalline nature leads to grains and grain boundaries that can strongly influence local defect transport and switching.^14–16^ Recent studies have demonstrated gradual and controllable conductance modulation in Y_2_O_3_-based memristors, making them attractive for analog computing and neuromorphic applications.^10,17–19^ Although transport models incorporating oxygen-vacancy dynamics have been developed to describe such analog behavior in filamentary RRAM,^20–25^ its microscopic origin and relationship to the underlying microstructure remain under debate, largely because filament evolution during gradual switching has not been directly visualized at the relevant scales.

While circuit-level constraints such as series resistance have been extensively considered in scaling memristor arrays, the nanoscale filament evolution during switching becomes increasingly critical upon downscaling. Conductive atomic force microscopy (c-AFM) provides a unique platform for locally addressing individual switching while simultaneously mapping their electrical response with nanoscale resolution. State-of-the-art c-AFM studies have enabled filament tomography through scalpel AFM^26–28^ and nanoscale investigations of filament formation.^14,15,29–33^ However, these approaches have primarily focused on post-mortem analysis, forming, or single-cycle observations,^34,35^ because the overhead current in LRS can substantially modify the c-AFM tip and sample. Consequently, direct *in situ* visualization of gradual filament evolution during repeated switching remains experimentally challenging.

Here we directly visualize and quantify nanoscale filament evolution in Y_2_O_3–*x*_ thin films using *in situ* c-AFM. Through controlled local switching, we resolve individual filament evolution during gradual SET and RESET processes and correlate their behavior with the underlying grains and grain boundaries. Multicycle and single-point switching experiments further reveal the dominant filament behaviors and local operation conditions, providing design guidelines for the polycrystalline ultradense crossbar array.

The substoichiometric Y_2_O_3–*x*_ films were grown by molecular beam epitaxy (MBE) with a constant evaporation rate of 0.7 Å/s while varying the oxygen radical flux, from 0.01 standard cubic centimeter (sccm) (most oxygen-deficient) to 0.2 sccm (most oxidized), to achieve a precise controlled broad oxidation range. The films were grown to a nominal thickness of 10 nm, as confirmed by X-ray reflectometry (XRR) measurements. In Y_2_O_3–*x*_ thin film studies, the crystal structure, monoclinic or cubic, is determined by the oxidizing conditions.^17,37,38^

In Figure 1a, the 2θ/ω measurements show a predominant cubic (222) reflection at an angle of 29.03° for all four distinct stoichiometries. At the highest oxygen flux, 0.2 sccm, a minor shoulder emerges next to the cubic reflection at approximately 30.4°, which can be attributed to the monoclinic (402̅) reflection, indicating a fully stoichiometric layer. With decreasing oxygen flux, the monoclinic feature gradually reduces until it disappears at a flow of 0.01 sccm. This trend further stabilizes the oxygen-deficient cubic phase, which inherently exhibits a defect-rich structure attributable to 1D oxygen vacancy channels.^10,17^

**Figure 1:**
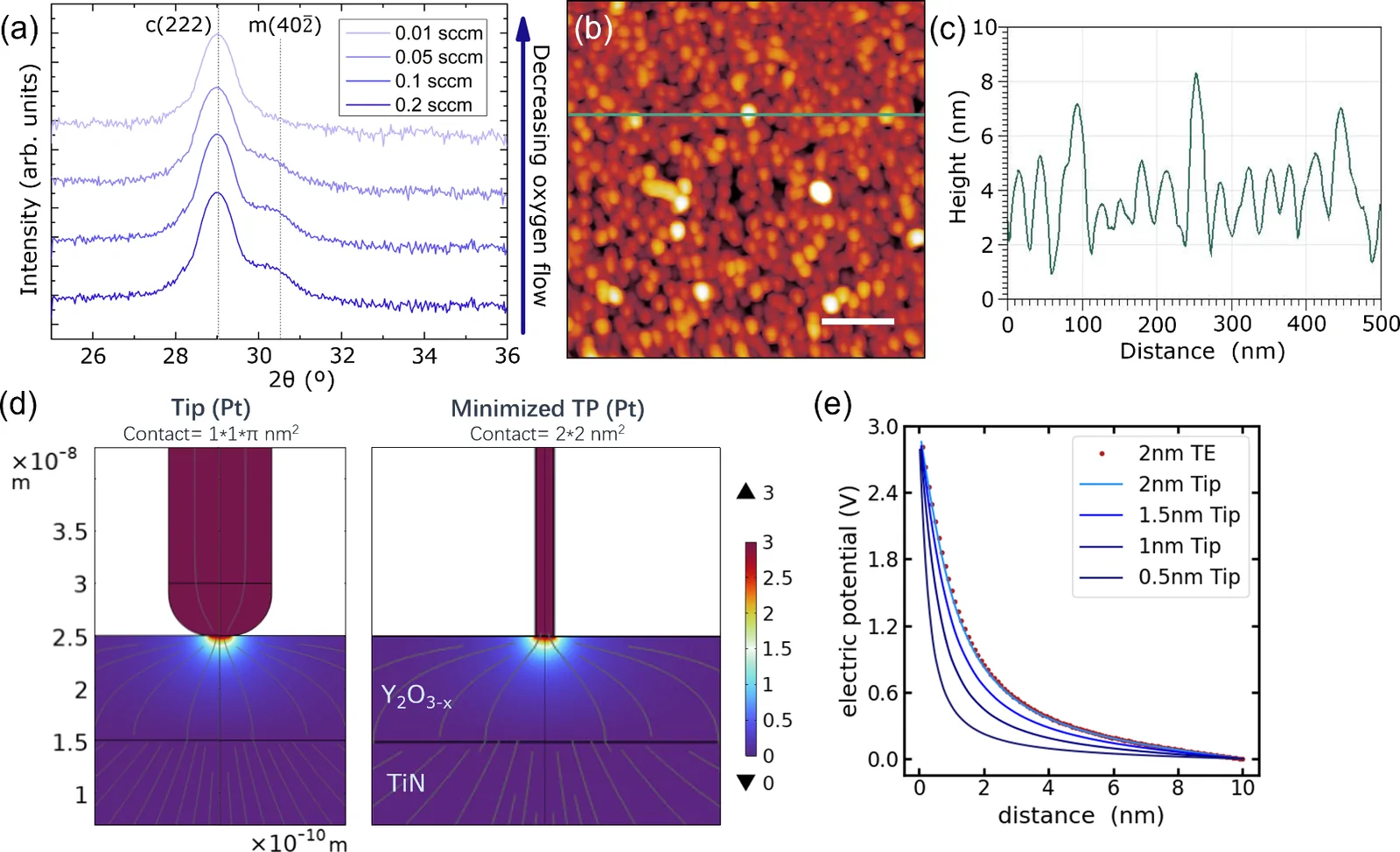
Substoichiometric Y_2_O_3–*x*_ thin film characterizations and tip–electrode electrical field simulation. (a) XRD measurements on the substoichiometric Y_2_O_3–*x*_ sample series. (b) AFM topography of the 0.01 sccm sample showing an RMS roughness of 1.5 nm. The white scale bar is 100 nm. (c) Height profile of the green line in (a). (d) COMSOL model of c-AFM tip versus 2 nm × 2 nm TE. Both cases with Pt as tip/electrode material and 3 V applied to the tip/electrode. (e) Potential gradient cross section under 2 nm × 2 nm TE and different circular contact areas of the c-AFM tip.

The topography of the most oxygen-deficient film, 0.01 sccm, is shown in Figure 1b, where grains and grain boundaries are visible. All samples exhibit a low root-mean-square (RMS) roughness of 1.5 ± 0.1 nm over 500 nm × 500 nm area, as shown in Figure S1. The line profile of the green line in Figure 1b is plotted in Figure 1c, showing the grain profiles. Except for the two higher peaks corresponding to grains initiating growth of the subsequent layer, most of the height variations are within 2–3 nm and 20–30 nm in width, in good agreement with the polycrystalline Y_2_O_3–*x*_ grain size observed using scanning transmission electron microscopy (STEM).^17,39,40^

The c-AFM tip, functioning as a nanoscale mobile top electrode (TE), may generate an electric-field distribution distinct from that of a conventional planar memristor electrode. However, as crossbar devices are scaled toward the nanoscale, for instance, with 2 nm-wide TEs,^41^ the electrode dimensions become comparable to the tip apex. To assess the applicability of the c-AFM tip as a nanoscale TE, we simulated the electric-field distributions of a 2 nm × 2 nm planar TE and a c-AFM tip with a 5 nm radius of curvature (ROC), incorporating circular contact areas with diameters of 0.5, 1, 1.5, and 2 nm using COMSOL Multiphysics. The corresponding geometries are illustrated in Figure 1d, and their calculated cross-section potential gradients are plotted in Figure 1e. Notably, the potential-gradient profiles of the 2 nm TE and 2 nm diameter tip contact, plotted as red dots and a light-blue curve, respectively, closely overlap. This close agreement indicates that the c-AFM tip provides a reasonable approximation of a nanoscale mobile TE under the present biasing conditions. In the following experiments, the tip locally actuates switching at each point through biased raster scanning, providing an analogue of nanoscale TE while enabling direct investigation of local switching phenomena.

A series of threshold voltage measurements across all samples is presented in Figure S2, revealing a systematic increase in the threshold voltage from approximately −1.4 to −4.8 V with increasing oxygen stoichiometry. This dependence is consistent with the field-driven vacancy dynamics reported for large-area Y_2_O_3–*x*_ systems,^17–19,42^ where a higher oxygen vacancy concentration lowers the nucleation threshold and facilitates filament formation at lower voltages. The particularly low threshold voltage of 1.4 V for the most oxygen-deficient sample (0.01 sccm) is of interest for low-power operation, so the investigation of nanoscale switching behavior is based on this substoichiometry.

To visualize the filament evolution at a nondestructive read voltage, we subsequently imaged the switched state at +0.25 V following each SET process of −2, −3, and −3.5 V. The gradual formation of conductive filaments at the respective SET voltages is shown in Figure 2a–c. When −2 V is applied to the area and read with +0.25 V, current appears between the grains in Figure 2a. This implies that filaments start to evolve from the grain boundaries, which aligns with the higher concentration of oxygen vacancies at the grain boundaries causing defect states in the local band gap.^14–16,46,47^ The larger spots, main current contributors, have a full width at half-maximum (fwhm) diameter of around 12–15 nm, while the smaller filaments have fwhm diameters of 5–8 nm, as analyzed in Figure S3.

**Figure 2:**
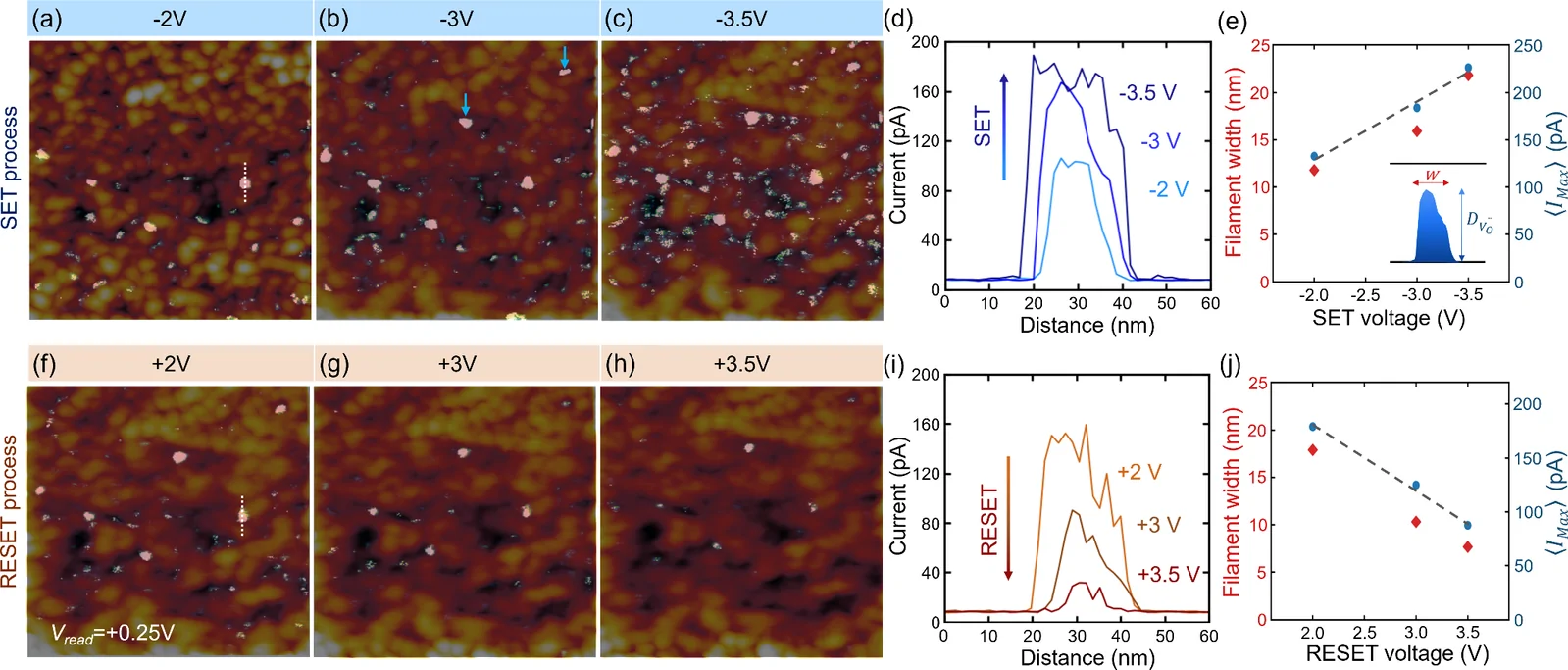
Topography and current map of incremental (a–e) SET and (f–j) RESET procedures. All images are 400 nm × 400 nm, and the current maps are measured at *V*_read_ = +0.25 V. The brighter parts show current levels exceeding 100 pA. (a–c) SET process with sequentially incremented voltages of −2 V, −3 V, and −3.5 V, respectively, followed by a readout at +0.25 V. (d) Current profile of the filament marked with the white dashed line in (a) after different SET processes. (e) Filament width and current correlation with the SET voltages. (f–h) RESET process with sequentially incremented voltages of +2 V, +3 V, and +3.5 V, respectively, followed by a readout at +0.25 V. (i) Current profile of the same filament marked with the white dashed lines in (a) and (f) at different RESET processes. (j) Filament width and current correlation with the RESET voltages.

After applying −3 V, as shown in Figure 2b, more current contributions appear compared to the −2 V SET condition. The pre-existing filaments expand slightly in diameter, while the emerging conducting points are generally smaller in size, except for the two relatively larger ones indicated by the blue arrows. After applying −3.5 V, as shown in Figure 2c, the number of larger conductive points remains the same, while the number of smaller points clearly increases. These smaller conducting points exhibit a homogeneous appearance along the grain boundaries.

Figure 2d,e further quantifies the evolution of the filament, marked with white dashed line in Figure 2a, during the SET process. With increasing SET voltage, the filament progressively grows as illustrated in Figure 2d. The current profiles are sharper on the left and smoother on the right, possibly due to c-AFM artifacts from the tip sidewalls. Nevertheless, this would not affect the overall trend of filament morphology as it remains a constant factor. The filament’s fwhm diameter during the SET process, read at +0.25 V, increases from 11.8 nm (upon −2 V) to 15.9 nm (upon −3 V) and 21.8 nm (upon −3.5 V). Concurrently, the maximum mean current, ⟨*I*_max_⟩, which averages the points above the half-maximum, increased from 133 pA (after −2 V) to 184 pA (after −3 V) and finally 226 pA (after −3.5 V). The diameter and current corresponding to the SET voltages are summarized in Figure 2e, exhibiting approximately linear dependence.

When applying inverse biases of +2 V, +3 V, and +3.5 V, the number of conductive spots gradually decreases, as seen in Figure 2f–h. After resetting the area with +2 V (Figure 2f), the smaller filaments reduced significantly, while the larger ones remained but shrank slightly. The total conducting area returns to a similar level to the −2 V SET shown in Figure 2a. After +3 V, the main larger filaments constrict moderately, as shown in Figure 2g, and the smaller filaments nearly vanish. After resetting with +3.5 V (Figure 2h), the main filaments narrow significantly and barely contribute to conductivity across the entire area.

During the incremental RESET procedure, the filament number, size, and current show a significant and systematic reduction. After resetting with +2 V, the filament diameter decreases from 21.8 to 17.9 nm, and the average current drops to 179 pA. Upon resetting with +3 V, the filament diameter shrinks to 10.3 nm with corresponding ⟨*I*_max_⟩ = 125 pA. This is smaller than that at the initial −2 V SET. After a +3.5 V RESET, the filament nearly disappears, appearing only 7.7 nm with ⟨*I*_max_⟩ = 87.5 pA. These RESET analyses are summarized in Figure 2j, where both filament diameter and current decrease linearly with RESET voltage, mirroring the linear behavior observed during SET.

The polarity-dependent SET and RESET behavior can be linked to the nanoscale redistribution dynamics of oxygen vacancies. Under negative tip bias, oxygen vacancies, V_O_^··^, migrate toward the surface, facilitating filament formation for SET, whereas positive tip bias redistributes V_O_^··^ toward the TiN bottom electrode, as RESET. A linear dependence of both the filament diameter and the corresponding current on the applied voltage is observed in both the SET and RESET processes. This indicates that the resistance is governed by both the filament width and the oxygen vacancy density, *D*_V_O_^··^_, within the conductive path. The ⟨*I*_max_⟩ reflects the electron density related to a given oxygen vacancy concentration. A schematic illustration of the filament shape is provided in the inset of Figure 2e. Note that the oxygen vacancy valence is not necessarily to be +2, however, V_O_^··^ is used to keep the Kröger–Vink notation for simplicity.

After a −2 V SET and readout at +0.25 V, multiple conductive channels have already appeared, indicating that the effective SET voltage lies below −2 V. This agrees well with the threshold voltage of the 0.01 sccm sample (approximately −1.4 V) extracted in Figure S2f. In conventional resistive switching devices, the LRS is typically dominated by one or a few highly conductive filaments within the device area, and the forming voltage is typically much higher than the SET voltage. The comparable values observed here therefore suggest forming-free nature in Y_2_O_3–*x*_, consistent with oxygen-deficient yttria-based RRAM,^17^ where the SET and forming voltages are in the range of −1.5 to −2 V.

While the larger filaments show widening and narrowing under controllable SET and RESET processes, additional smaller filaments emerge only at higher voltages. In an ultradense memristor array system, individual devices may be confined to regions lacking these dominant filaments because of the spatially heterogeneous defect landscape, potentially requiring higher programming voltages to achieve sufficient filament density.

Therefore, we performed multicycle, full-area switching at elevated SET/RESET voltages to assess switching uniformity and to further elucidate the persistence and dominance of individual filaments during repeated operation. In this series, the initial topography (Figure 3a) and the current map (Figure 3b) are acquired simultaneously at *V*_read_ = +0.5 V. In the pristine state, there is merely any current, except for the two brighter spots. The SET process is performed by scanning over the area at −5 V, followed by reading the conductivity using *V*_read_ = +0.5 V. Figure 3c shows that the first SET (LRS#1) surface has plenty of conducting points, including both large and small filaments. After a +5 V RESET, the first high-resistance state (HRS#1) exhibits no current above 30 pA (Figure 3d). The lower current in the HRS compared to the pristine state has also been observed in yttrium oxide memristors.^17^ In the second cycle, LRS#2 again shows multiple conductive sites (Figure 3e), though compared to that in LRS#1, the larger conducting points shrink while many weaker filaments disappear. Following the second RESET, HRS#2 (Figure 3f) again shows no measurable current above 30 pA.

**Figure 3:**
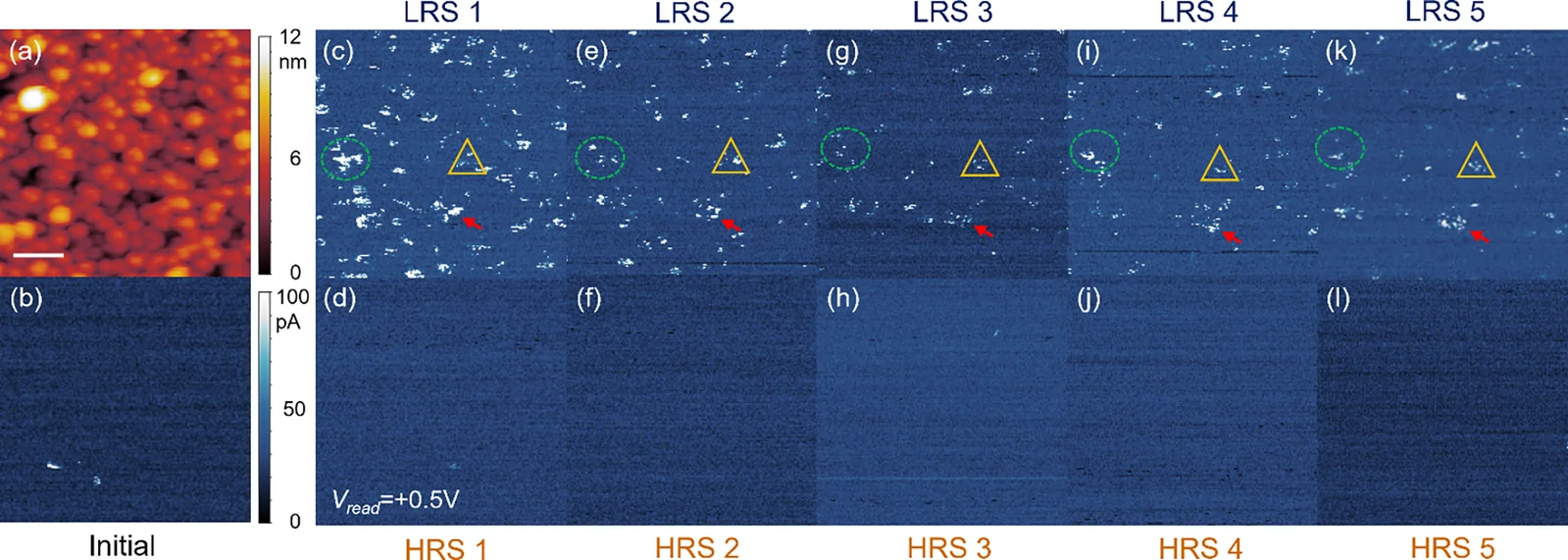
Multicycle switching in the same area using −5 V (SET), +5 V (RESET), and +0.5 V (read). (a) The initial topography of the area and (b) its corresponding current map. (c–l) Upper panel: LRS obtained after SET operations #1–5. The marks indicate persistent conducting regions. Lower panel: HRS obtained after RESET operations #1–5. All current images were read out using +0.5 V. The white scale bar in (a) is 50 nm.

Across repeated cycles from LRS#1 (Figure 3c) to LRS#5 (Figure 3k), the overall current gradually decreases. This trend agrees with the observed gradual SET/RESET that the RESET requires a lower absolute voltage than the SET. Notably, the conductive paths persisting in LRS#5 correspond to the larger filaments formed during LRS#1 (see marks in Figure 3 (c-k)). This suggests that subtle nanoscale stoichiometric variations, which induce early stage filament formation, govern subsequent switching cycles. To reach higher filament density, operation would likely require increased bias, implying enhanced switching-voltage variability at extreme scaling. On the other hand, through grain size engineering, the number of grain boundaries, where the filaments predominantly form, can be utilized to ensure higher filament forming probability and thus low-power operation The results above are based on full-area raster scanning, however, in an ultradense crossbar array, individual memristors are spatially confined and must switch independently despite their close proximity. To address this, independent single-point switching was investigated by sweeping bias at fixed locations without raster scanning. This configuration serves as a nanoscale analogue of independently addressed array cells and enables evaluation of potential crosstalk or interference between neighboring regions. A selected zoom-in region is first imaged, as shown in Figure 4a, after which the tip is relocated to specific positions, intentionally at the grain boundaries, for single-point voltage sweeping. The SET process is performed by sweeping the tip bias from 0 V to −6 V, while RESET is achieved through a full-area scan at tip bias *V*_tip_ = +3 V. To further highlight nanoscale memristor behavior, *I*–*V* measurements are conducted by a voltage sweep sequence of 0 V → −6 V → +5 V → 0 V, using a step size of 10 mV and dwell and integration time of 50 ms.

**Figure 4:**
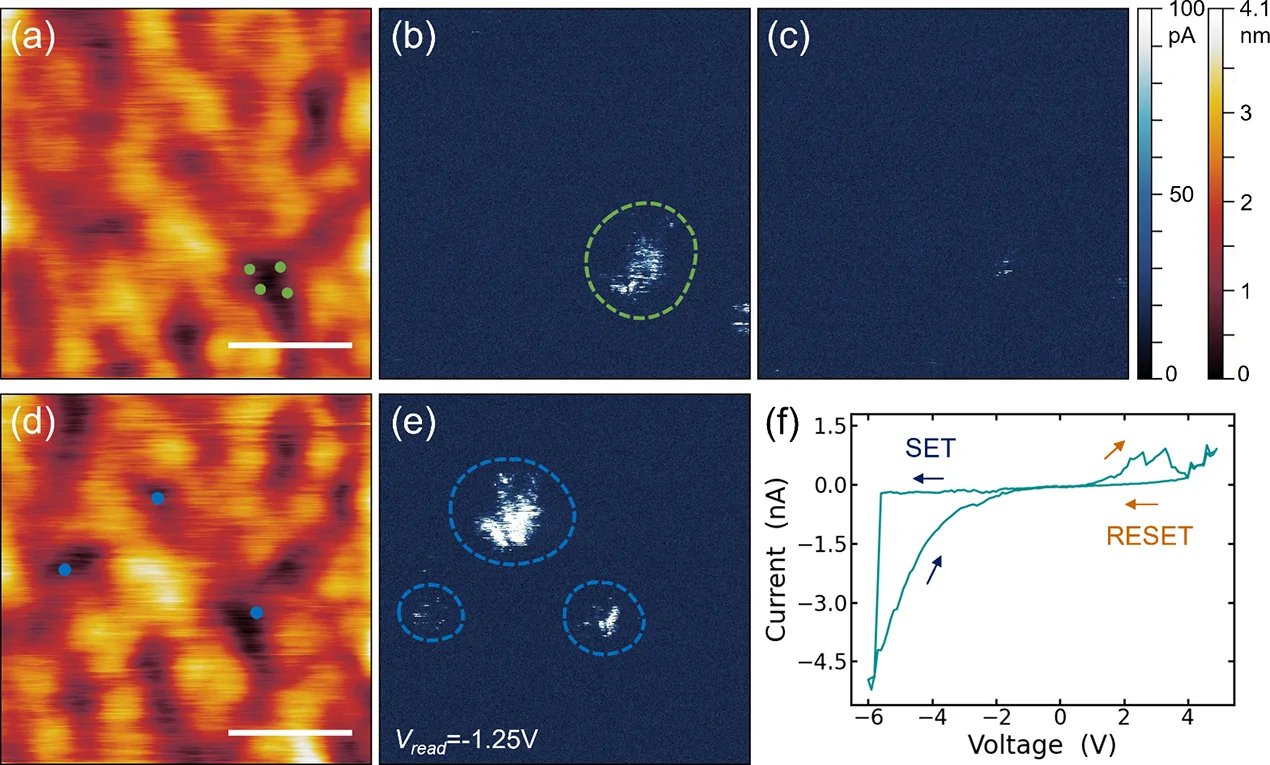
Single-point resistive switching and single-point I–V sweep. (a) Initial state topography. (b) Current map of the area of (a) upon the individual −6 V SET applied at the four green points marked in (a), and read out at tip bias −1.25 V. The green dashed circle highlights the current arising from the SET process. (c) The current map after the +3 V full-area RESET process and read at −1.25 V. (d) Topography after SET and RESET operations. The three blue dots mark spatially separated sites for the next individual switching cycle. (e) The current map measured upon the three individual single-point SET processes, with the induced currents highlighted by the blue dashed circles. The white scale bars are 50 nm. (f) Single-point IV switching at a grain boundary.

The initial topography and its corresponding current map are shown in Figures 4a and S5b, respectively, where no conductive spots are detected. After applying SET at four nearby positions (green dots in Figure 4a), a conductive region emerges in Figure 4b, highlighted by the green dashed circle. Notably, this conducting area can subsequently be erased by a +3 V RESET scan across the entire region, as shown in Figure 4c. The observed conductive region, extending laterally over 20 nm, may originate from the cumulative effect of the four individual SET processes as well as from the intrinsic lateral extent of a single filament.

To isolate independent switching, three points separated by 45–80 nm were selected (indicated in Figure 4d) and subjected to independent SET operations at −6 V. A slight positional drift is observed in Figure 4d relative to Figure 4a due to AFM piezo drift. Nevertheless, the structural features remain clearly recognizable. Markedly different current responses are observed in Figure 4e despite identical electrical processes being applied. This divergent current response at *V*_read_ = −1.25 V, below the threshold voltage, probes relative local resistance at each point, reflecting local V_O_^··^ distribution. More importantly, the lateral influence of a single-switching event can extend up to ∼20 nm, as shown in Figure S4, indicating that electrical crosstalk between neighboring devices may become significant when memristor spacing approaches this length scale. This range falls within the regime of thermal crosstalk in a highly integrated array with memristor spacings on the order of a hundred nanometers.^48^ However, through microstructure engineering, the lateral size of the conducting region can be adjusted to mitigate these crosstalk effects.

Single-point switching requires a higher SET voltage. Even at a relatively high bias of −6 V, some locations do not exhibit a strong current response, although the threshold voltage for full-area switching being below −1.5 V. This behavior arises because, during single-point SET operations, a confined electric-field beneath a nanometer-scale tip, without cumulative effect, encompasses fewer defect-rich regions where filaments preferentially form. Therefore, nanometer-sized TEs require a higher potential to drive local V_O_^··^ migration.

An *I*–*V* sweep at a single point was performed at a nearby grain boundary, as shown in Figure 4f. Pronounced hysteresis characteristics of memristive switching are observed, including a sharp SET process above −5 V and a moderate RESET process around +2.5 V. The relatively high operation voltages observed with a fixed c-AFM tip further confirm that when the TE approaches the c-AFM tip apex dimension, <5 nm, higher voltages are required. Overall, the observed behavior is consistent with the scanning results, while quantitative parameters may vary due to spatial inhomogeneity in oxygen stoichiometry. The relatively high voltages were intentionally applied to ensure reliable detection of conductive events in the c-AFM measurements.

From the design perspective, each minimized memristor cell in a crossbar array should therefore include at least part of a grain boundary. Hence, the grain diameter defines the scaling limit of the crossbar device area. The equivalent area radius *R*_eq_ of the Y_2_O_3–*x*_ films ranges from 5 to 7 nm, independent to oxygen flow (Figure S1). Further scaling while maintaining a reasonable threshold voltage would require grain-size engineering in other viable methods, for example, growth temperature, evaporation rate, or bottom electrode selection.

Our findings describe filament evolution in polycrystalline thin films and are broadly applicable to any technique producing a comparable microstructure. In particular, sputtering, widely used in industrial manufacturing, yields polycrystalline films with tunable oxygen stoichiometry and is therefore directly relevant. Atomic layer deposition (ALD) may produce amorphous films for certain material systems, appropriate growth conditions can also yield polycrystalline microstructures, in which grain-boundary-mediated filament evolution may likewise become relevant.

In summary, we have demonstrated reliable nanoscale resistive switching and gradual SET and RESET behavior in Y_2_O_3–*x*_ thin films using c-AFM, providing a route toward controllable multilevel states in scaled memristors. Systematic *in situ* c-AFM measurements reveal a clear correlation between oxygen stoichiometry and the switching threshold, which can be tuned from approximately −1.4 V to −5 V. Filaments preferentially initiated at grain boundaries can be linearly modulated in width and *D*_V_O_^··^_ through applied biases, enabling analog switching. Multicycle measurements further show that the dominant active filaments correspond to those with larger diameters established during the first SET process.

Single-point switching experiments, together with the electrical field simulations, demonstrate that a c-AFM tip can function as a nanosized mobile TE, enabling localized switching and direct investigation of nanoscale memristor operation. The influence of an individual switching event extends laterally over approximately 20 nm, providing an experimentally determined length scale relevant to device-to-device spacing and potential crosstalk in highly integrated arrays. The minimum device area, grain-size dependent, requires substantially higher operating voltages for reliable switching.

Together, these results establish direct links between oxygen stoichiometry, microstructure, filament morphology, and nanoscale switching behavior. These observations provide new insight into the microscopic physics of filamentary resistive switching and establish c-AFM as a powerful approach for bridging nanoscale filament dynamics with device-level switching.

## Supplementary Material



## Notes

Experimental section, including sample preparation, XRD, XRR, vacuum transfer, c-AFM, and potential distribution simulations; topography and grain analysis of substoichiometric Y_2_O_3–*x*_ films; threshold voltage investigation for the substoichiometric series; conductive filament current profile analysis; multicycle single-point switching (PDF)
