# Supplementary material for: Nanoscale Filament Evolution and Local Resistive Switching in Substoichiometric Yttrium Oxide Thin Films
Source: Nano Lett. 2026 Sep 28;26(42):14794–801. doi: 10.1021/acs.nanolett.6c04262 (PMC13721146; doi:10.1021/acs.nanolett.6c04262)
Supplement: Supplementary file 1 [file nl6c04262_si_001.pdf]

NANO LETTERS

Supporting Information for

Nanoscale Filament Evolution and Local Resistive Switching in Sub-Stoichiometric Yttrium Oxide Thin Films

Yen-Po Liu[1], Erkai Wang[1], Philipp Schreyer[2], Yu Duan[2], Luisa Bayer[1], Yingxin Li[2], Taewook Kim[2], Alexey Arzumanov[2], Stefan Wiefels[1], Eszter Piros[2], Lambert Alff[2], Regina Dittmann[1]

[1]Peter Grünberg Institute (PGI-7), Forschungszentrum Jülich GmbH, 52425 Jülich, Germany

[2]Advanced Thin Film Technology Division, Institute of Materials Science, 64287 Darmstadt, Germany

Table of Contents:

- Experimental section (Sample Preparation, XRD, XRR, vacuum transfer, c-AFM, Simulations)
- Topography and grain analysis of sub-stoichiometric Y_2O_{3-x} films
- Threshold voltage investigation for the sub-stoichiometric series
- Conductive filament current profile analysis
- Multi-cycle single-point switching

Table of Figures:

- **Figure S1.** Topography and grain analysis of sub-stoichiometric Y_2O_{3-x} films.
- **Figure S2.** Threshold voltage investigation for the sub-stoichiometric series.
- **Figure S3.** Current profiles of the conductive filaments.
- **Figure S4.** Single-point switching: filament current profile analysis.
- **Figure S5.** Multi-cycle single-point switching.

- **Experimental section**

Sample Preparation

The deposition of thin films was carried out using a custom-designed reactive MBE with a base pressure of ~10^{-8} mbar. All samples were deposited on 10×10 mm c-cut sapphire substrates and mounted on a specially designed sample holder to have good electrical contacts for c-AFM measurements. TiN bottom electrodes were grown at a substrate temperature of approximately 450 °C, with an evaporation rate of 0.05 Å/s from a metallic Ti source. Nitrogen gas was flowing at a rate of 1 sccm, and a controlled flux of N radicals was achieved through a radio-frequency (RF)-activated plasma at 320 W. The temperature was regulated via thermocouple-controlled resistive heating. Y_2O_{3-x} layers with differing stoichiometries were then grown *in-situ* at the same temperature. The evaporation rate of metallic Y was maintained at a constant rate of 0.7 Å/s, while the flow of oxygen radicals was modulated within the range of 0.2 to 0.01 sccm at an RF power of 200 W. In-situ transfer is described in the SI.

X-ray Diffraction and Reflectometry

X-ray diffraction and reflectometry measurements (XRD, XRR) were conducted utilizing a Rigaku SmartLab four-axis goniometer equipped with a rotating Cu anode. All samples were characterized in a parallel beam configuration, using a Ge(220) double-bounce monochromator that utilized Cu Kα1 radiation. The thickness of the TiN was calibrated to 30 nm via XRR measurements, and the thickness of Y_2O_{3-x} was calibrated to 10 nm, respectively.

Sample Transfer

In order to prevent oxidation of the sub-stoichiometric Y_2O_{3-x} layers, the samples were kept in ultra-high vacuum throughout the whole process. In this regard, a custom-designed vacuum suitcase by Mantis Deposition Ltd. (Birmingham, UK) was employed for the transfer of the samples from TU Darmstadt to the Scanning Probe Microscopy (SPM) chamber at Forschungszentrum Jülich GmbH.

Conductive Atomic Force Microscopy

The c-AFM is performed using Omicron VT-SPM. Bruker conductive diamond probes, AD-2.8-SS, are used in this work. The total measured resistance consists of contributions from the thin film, the conductive tip coating, and the internal resistance of the measurement system, while the latter two remain constant. The scanning parameters are setpoints 1 nN for scanning and 1.5-2 nN for single point switching, and a scanning speed 200 nm/s. Electrical *IV* sweep were performed using MATRIX SPM control with step size 10 mV and waiting and integration time 50 ms. To prevent unexpected current spikes that could locally burn the film and damage the diamond tip, a current compliance was applied to the SPM ground using a Keithley 2400 sourcemeter.

Potential Distribution Simulations

Simulations of the potential and electric-field distributions in the tip–sample and comparative nanoelectrode junctions were performed using COMSOL Multiphysics Version 6.4. The model consisted of a conductive probe or nanoelectrode, 10 nm-thick Y_2O_3 film, and 30 nm-thick TiN

layer. For the tip-sample junction, the probe was modeled as a 10nm-diameter cylindrical diamond body and a rounded apex. The tip-Y_2O_3 contact was defined as a circular area with diameters of 2.0, 1.5, 1.0, 0.5 nm. The lateral dimensions of the Y_2O_3/TiN stack are specified as 100 x 100 nm^2. For comparison, a Pt nanoelectrode with a lateral dimension of 2×2 nm^2 and a height of 50 nm was modeled on the same Y_2O_3/TiN stack. The material densities used in the simulation are ρ_{TiN} = 5400 kg/m^3, $\rho_{Y_2O_3}$ = 4900 kg/m^3, ρ_{Diamond} = 3530 kg/m^3 and ρ_{Pt} = 21450 kg/m^3. The corresponding electronic conductivities were set to σ_{TiN} = 7.41×10^5 S/m, $\sigma_{Y_2O_3}$ = 1.0×10^{-3} S/m, $\sigma_{Diamond}$ = 3.33×10^5 S/m (Bruker AD-2.8-SS specification), and σ_{Pt} = 9.43×10^6 S/m were used.

▪ Topography and grain analysis of sub-stoichiometric Y_2O_{3-x} films

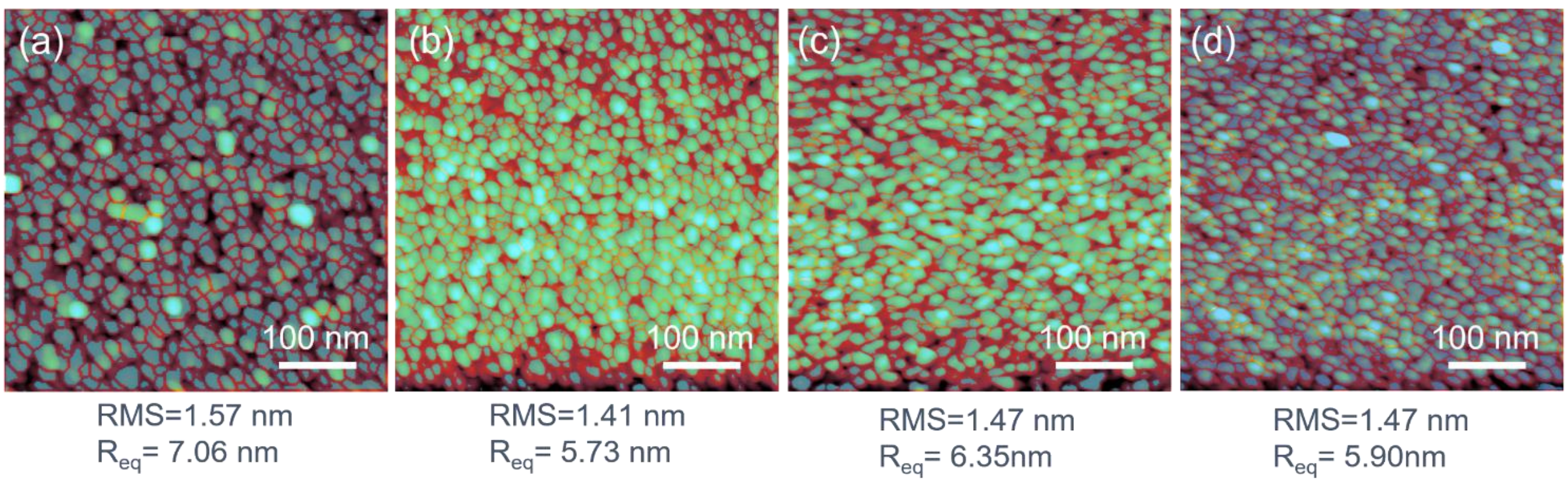

Figure S1. Topography and grain analysis of sub-stoichiometric Y_2O_{3-x} films grown with different oxygen flows (a) 0.01 sccm, (b) 0.05 sccm, (c) 0.1 sccm, and (d) 0.2 sccm. These figures show grain-segmented maps with the corresponding surface RMS roughness and the equivalent area radius R_{eq}.

▪ Threshold voltage investigation for the sub-stoichiometric series

Simultaneous bias-dependent current maps of the 0.01 sccm sample acquired over the same surface area reveal progressive activation of localized conduction sites as the tip bias, V_{SET}, increases from -0.25 V, -1.25 V, -1.5 V, to -2.0 V, shown in Figure S2(a-d). Brighter dots indicate the current response to the external bias, as conduction pathways, whereas the dark areas indicate negligible current response. The conductive area expands nonlinearly with bias, as can be seen from (a) to (d), indicating field-driven activation of defect-mediated transport. A superposition of the topography and the current map acquired at V_{SET}=-1.5V is illustrated in Figure S2(e), showing the correlation between emerging current and surface morphology.

The normalized conductive area of the 0.01 sccm sample as a function of applied biases is plotted in light blue in Figure S2(f). The threshold voltage is estimated by linear fitting to the three data points with a normalized conductive area above 0.02, yielding a threshold voltage of approximately -1.4 V. Increasing oxygen flux systematically shifts the threshold toward higher voltages. The 0.05, 0.1 and 0.2 sccm films exhibit threshold voltages of -2.9, -3.3, and -4.8 V, respectively, with corresponding data and linear fits shown in gray, yellow, and pink.

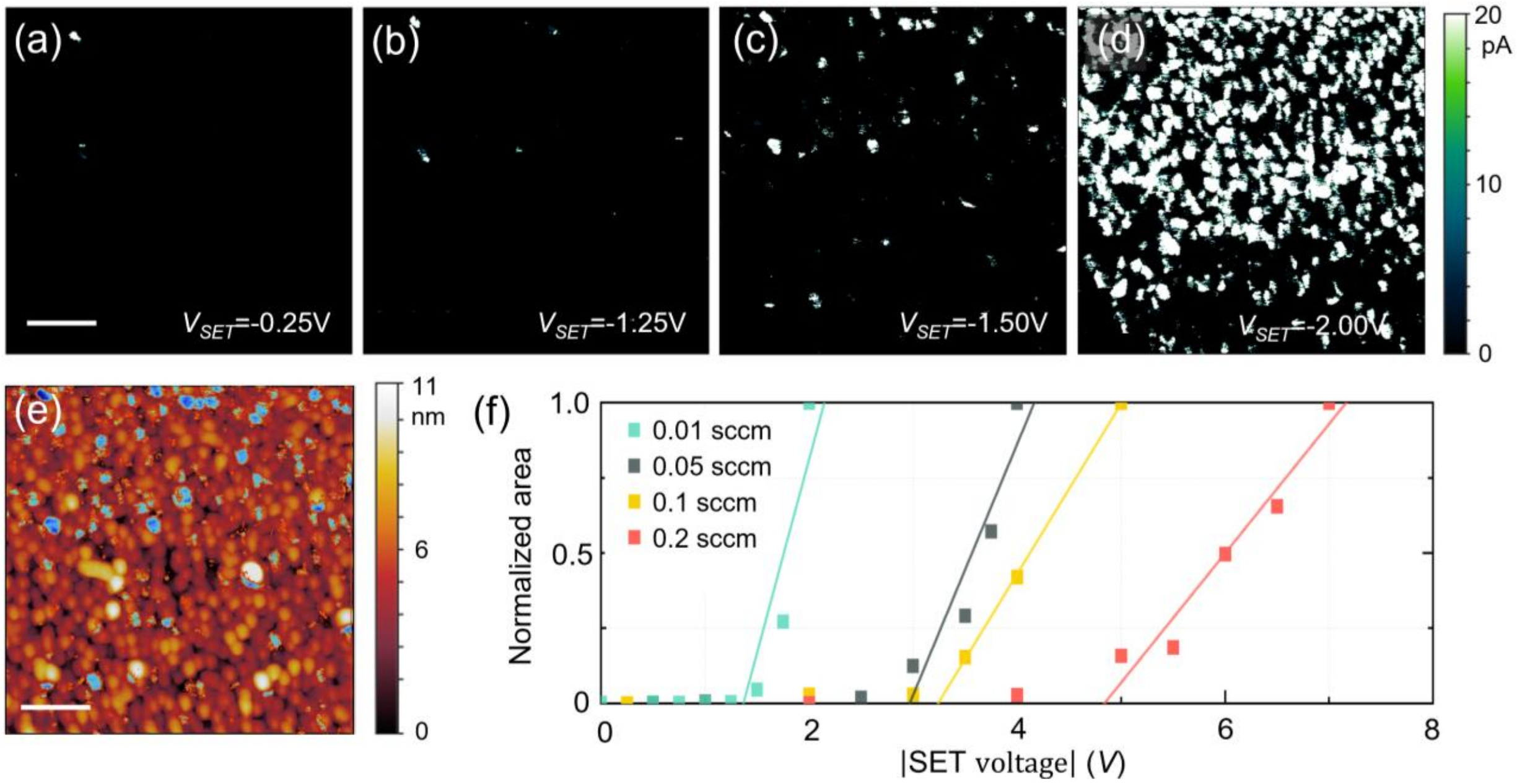

Figure S2. Threshold voltage investigation for the sub-stoichiometric series. (a-d) Current maps of the least oxidized sample corresponding to applied voltages from -0.25 V to -2 V, exhibiting increasing current from the surface area of (e). The white scale bar is 100 nm. (e) Superposition of the topography and the current map acquired at -1.5 V. Blue indicates the current above 20 pA. (f) Normalized area with a current above 2 nA for oxygen-deficient Y_2O_{3-x} samples with growth oxygen radical flow of 0.01 to 0.2 sccm. The intersection of the fit with zero baseline results in the threshold voltage.

Reliable threshold voltage extraction across all samples is enabled by current compliance control (using Keithley 2400 integrated c-AFM circuit), which suppresses current overshoot during forming and setting that cause modifications of the tip, its conductive coating, and film. When comparing the Y_2O_{3-x} thin films to HfO_x-based samples, it is found that the resistance is generally higher and that fewer current spikes are observed[1,2]. This indicates a more homogeneous distribution of oxygen defects over thin films. The detected low-current switching further points to energy efficient memristors.[3,4]

- ***Conductive filament current profile analysis***

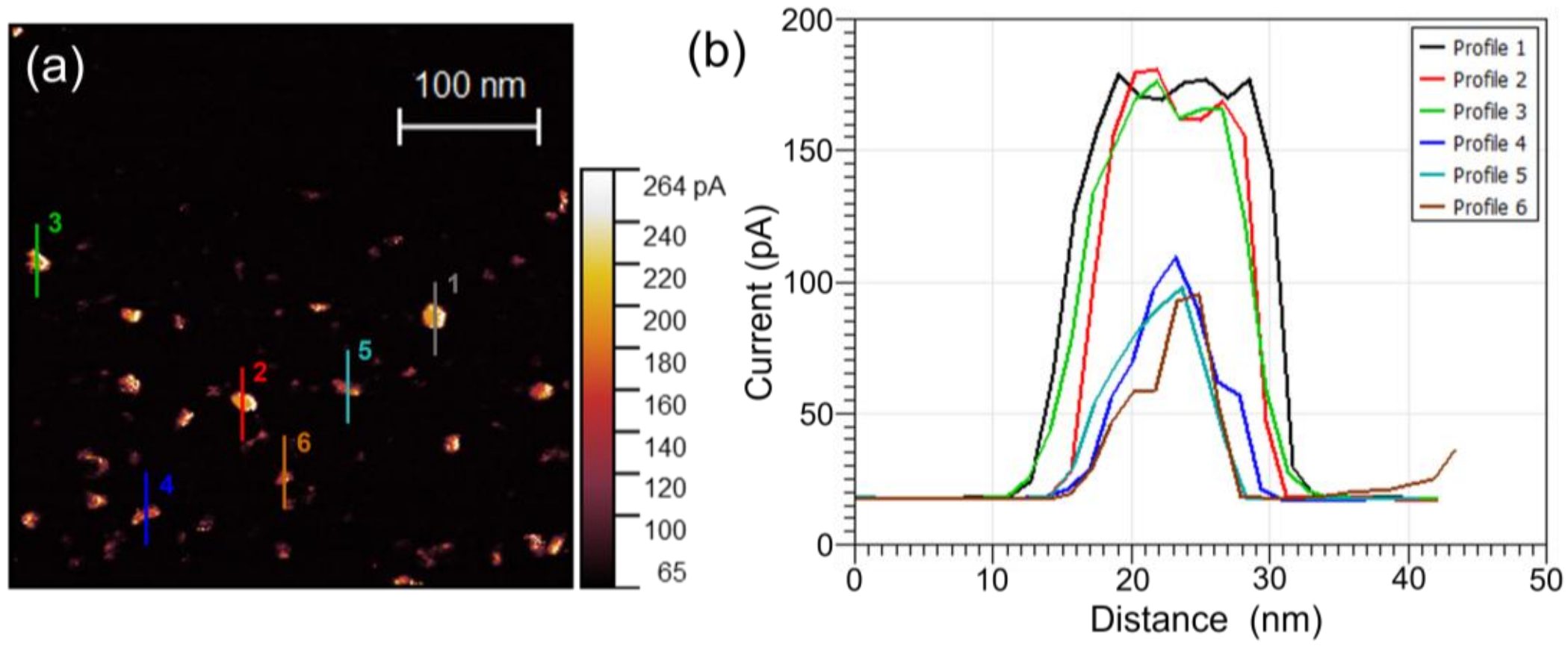

Figure S3. Current profiles of the conductive paths, showing the size and the current density of the filaments contributing to LRS.

The experimentally approximated diameters may be overestimated due to the tip convolution, which depends linearly on the tip radius. In contrast, the measured current is governed by the closest point of approach, with conduction mainly occurring through the tip apex. Consequently, tip-induced broadening in conductivity maps is generally weaker than in topographic images.

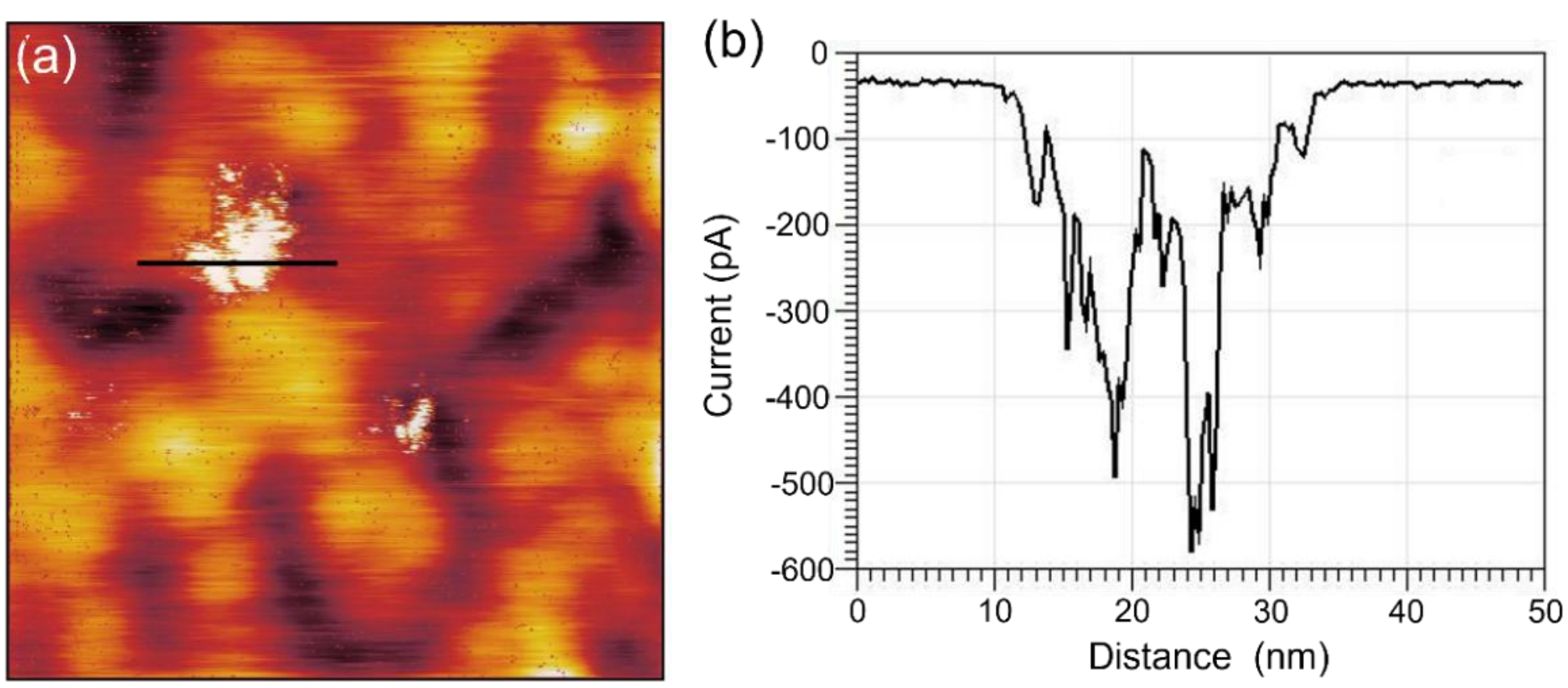

Figure S4. Single-point switching current profile. (a) superposition of topography and current map, with the brighter spot for current over 200 pA. The image is 150 nm by 150 nm. (b) current profile of the black line marked in (a).

- ***Multi-cycle single-point switching***

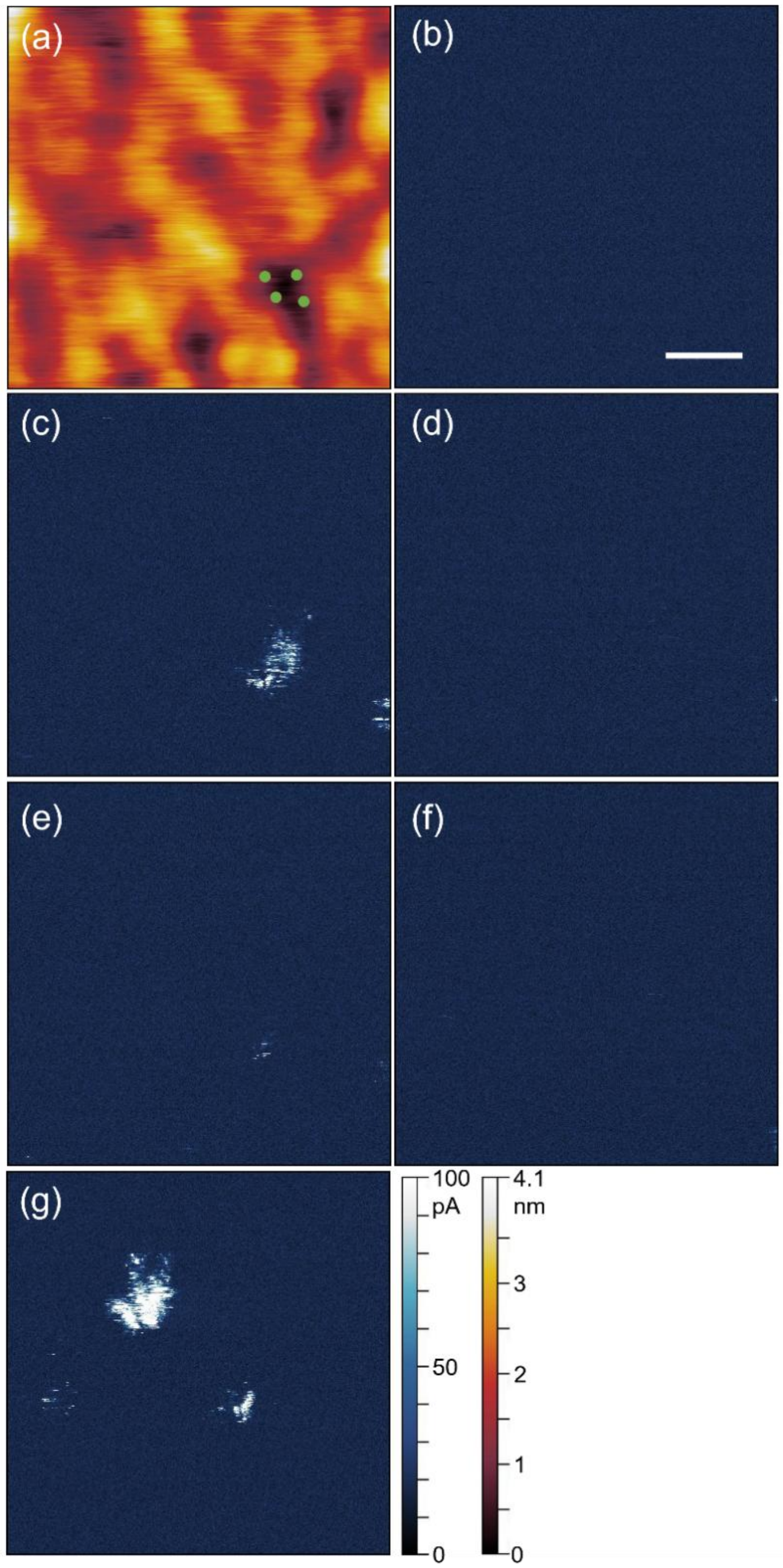

Figure S5. Multi-cycle single-point switching (a) Topography (b-g) current map of the area upon operations. (b) Initial state. The white scale bar is 30 nm. (c) After the SET process applied to the 4 green points marked in (a). (d) RESET process of +3 V applied over the full area. (e) SET process performed at a single point around the green dots. (f) The 2nd RESET scan over the area. (g) After the SET process applied to the 3 blue points marked in Figure 4(d).

References

(1) Schmidt, N.; Kaiser, N.; Tobias, V.; Piros, E.; Karthäuser, S.; Waser, R.; Alff, L.; Dittmann, R. Impact of Non-Stoichiometric Phases and Grain Boundaries on the Nanoscale Forming and Switching of HfOx Thin Films. *Adv. Electron. Mater.* **2024**, *10* (2300693).

(2) Kaiser, N.; Vogel, T.; Zintler, A.; Petzold, S.; Arzumanov, A.; Piros, E.; Eilhardt, R.; Molina-Luna, L.; Alff, L. Defect-Stabilized Substoichiometric Polymorphs of Hafnium Oxide with Semiconducting Properties. *ACS Appl. Mater. Interfaces* **2022**, *14* (1), 1290–1303. https://doi.org/10.1021/acsami.1c09451.

(3) Zhou, J.; Cai, F.; Wang, Q.; Chen, B.; Gaba, S.; Lu, W. D. Very Low-Programming-Current RRAM With Self-Rectifying Characteristics. *IEEE Electron Device Letters* **2016**, *37* (4), 404–407. https://doi.org/10.1109/LED.2016.2530942.

(4) Luo, Q.; Xu, X.; Liu, H.; Lv, H.; Gong, T.; Long, S.; Liu, Q.; Sun, H.; Banerjee, W.; Li, L.; Gao, J.; Lu, N.; Liu, M. Super Non-Linear RRAM with Ultra-Low Power for 3D Vertical Nano-Crossbar Arrays. *Nanoscale* **2016**, *8* (34), 15629–15636. https://doi.org/10.1039/C6NR02029A.
